# Addition of admission lactate levels to Baux score improves mortality prediction in severe burns

**DOI:** 10.1038/s41598-021-97524-9

**Published:** 2021-09-10

**Authors:** Ingrid Steinvall, Moustafa Elmasry, Islam Abdelrahman, Ahmed El-Serafi, Folke Sjöberg

**Affiliations:** 1 Department of Hand Surgery, Plastic Surgery and Burns, Linköping University, Linköping, Sweden; 2 Department of Biomedical and Clinical Sciences, Linköping University, Linköping, Sweden; 3 Medical Biochemistry department, Faculty of Medicine, Suez Canal University, 41522 Ismailia, Egypt; 4 Department of Anaesthesiology and Intensive Care, Linköping University, Linköping, Sweden

**Keywords:** Outcomes research, Risk factors

## Abstract

Risk adjustment and mortality prediction models are central in optimising care and for benchmarking purposes. In the burn setting, the Baux score and its derivatives have been the mainstay for predictions of mortality from burns. Other well-known measures to predict mortality stem from the ICU setting, where, for example, the Simplified Acute Physiology Score (SAPS 3) models have been found to be instrumental. Other attempts to further improve the prediction of outcome have been based on the following variables at admission: Sequential Organ Failure Assessment (_a_SOFA) score, determinations of _a_Lactate or Neutrophil to Lymphocyte Ratio (_a_NLR). The aim of the present study was to examine if estimated mortality rate (EMR, SAPS 3), _a_SOFA, _a_Lactate, and _a_NLR can, either alone or in conjunction with the others, improve the mortality prediction beyond that of the effects of age and percentage total body surface area (TBSA%) burned among patients with severe burns who need critical care. This is a retrospective, explorative, single centre, registry study based on prospectively gathered data. The study included 222 patients with median (25th–75th centiles) age of 55.0 (38.0 to 69.0) years, TBSA% burned was 24.5 (13.0 to 37.2) and crude mortality was 17%. As anticipated highest predicting power was obtained with age and TBSA% with an AUC at 0.906 (95% CI 0.857 to 0.955) as compared with EMR, _a_SOFA, _a_Lactate and _a_NLR. The largest effect was seen thereafter by adding _a_Lactate to the model, increasing AUC to 0.938 (0.898 to 0.979) (*p* < 0.001). Whereafter, adding EMR, _a_SOFA, and _a_NLR, separately or in combinations, only marginally improved the prediction power. This study shows that the prediction model with age and TBSA% may be improved by adding _a_Lactate, despite the fact that _a_Lactate levels were only moderately increased. Thereafter, adding EMR, _a_SOFA or _a_NLR only marginally affected the mortality prediction.

## Introduction

Outcome prediction models, such as mortality prediction, are essential tools when assessing the quality of care and outcome^1–3^. Furthermore, these techniques are often mandatory in the process of care improvement^1,2,4^. As early as the 1960’s, in the field of burns care, prediction of mortality has been based mainly on calculations that have used age and percentage total body surface area (TBSA%) burned, as described by Serge Baux^5^. The strong effect of age and burn size on mortality had, however, been appreciated and discussed as early as 1949^6^. Since the sixties, many attempts have been made to further improve the burn mortality prediction model^7,8^. The most successful model may be claimed to be that of Osler et al. who developed the revised Baux score by adding inhalation injury to age and TBSA% burned, and the Thermal Injury Mortality Model (TIMM) score which also uses nonlinear transformations as well as two-way interactions among the main effects to adjust for non-linearity and interactions of the variables^9^. Despite obtaining a significantly better prediction using the more complex model, the magnitude of this effect was less important clinically^9^. The models so far have been based, in most cases, on the whole cohort of burns and not on specifically those who needed critical care support, which is the most interesting group for mortality prediction. In the latter case, the variables of the Baux score have still performed well, but as the number of patients is decreasing over time, the distributions of the burn size range will get smaller along with the mortality rates, the prediction consequently gets less precise. This has been discussed by Steinvall et al. in a recent review^3^.

There is a further need to improve the outcome and especially mortality predictions for burns critical care settings. There are other well-known prediction models in general critical care medicine, not including burns, the most important of which are the Acute Physiology and Chronic Health Evaluation (APACHE IV) version 4^10^ and Simplified Acute Physiology Score 3 (SAPS 3)^11^. Both rely on patients’ characteristics and physiological data at admittance. It may be anticipated that APACHE IV or SAPS 3 may contribute to the burn outcome prediction model alone or when added to the variables of the Baux score. This has, however, not yet been examined. Furthermore recently, three other measures to improve the precision in critical care mortality outcome predictions have been presented and shown to be effective. These are, at admission: _a_Lactate^12^, Sequential Organ Failure Assessment score (_a_SOFA)^13^, and Neutrophil to Lymphocyte ratio (_a_NLR)^14^. Each of these scores or measures may be hypothesised to separately and/or in conjunction to the others improve the overall mortality prediction model also in burns who need critical care. The aim was to investigate whether these proposed scores and measures; either separately or in conjunction with each other, and when added to age and TBSA% burned, improved the mortality prediction among patients with severe burns who need critical care. Of interest was also whether this improvement would be clinically important.

## Methods

**Figure 1:**
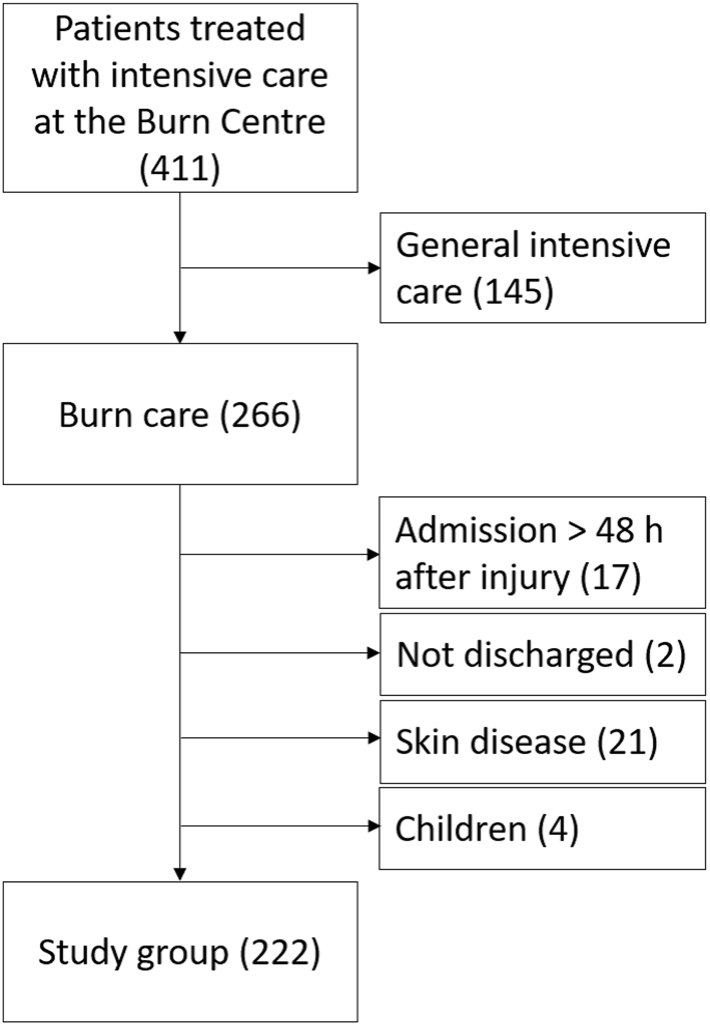
Flowchart over patient selection.

All patients with burns that required intensive care at the Burn Centre in Linköping University Hospital, Linköping, Sweden during 2010–2020 were recorded in the Swedish Intensive Care Registry (SIR). All recordings in the SIR of patients treated at the Burn Centre during 2010–2020 were retrieved. Patients who were treated for general intensive care (not burns) at the Burn Centre were excluded. Exclusion criteria for patients who were admitted for burn care were: admission later than 48 h after injury; those not discharged at the time of the study; and children age < 16 years (Fig. 1).

The definition of intensive care by SIR includes surveillance, diagnostics, treatment, and nursing of patients with an acute or life-threatening disease. Intensive care is carried out within a specific unit that meets certain basic requirements. These settings have recently been documented^15^.

The Burn Centre joined the Swedish Intensive Care Registry in 2010. Estimated mortality rate (EMR) was calculated using SAPS 3 for adult patients during the whole study period. Variables retrieved from SIR were: mortality; number of days under intensive care and on the ventilator at the Burn Centre; EMR; and _a_SOFA. Variables retrieved from the prospectively-maintained local burns’ registry^16,17^ were age, sex, the size and depth of the burn, and total duration of hospital stay at the Burn Centre. Admission laboratory values such as blood _a_Lactate concentration and _a_NLR were retrieved from the hospital’s medical records. Mortality was defined as death from any cause during the inpatient stay at the Burn Centre.

### Burn care

Adult patients who required intensive care were also later admitted to the inpatient ward at the Burn Centre during their hospital stay. In general, these patients had intensive care during the first weeks or months, followed by intermediate care, which included interventions under anaesthesia such as operations and repeated wound care procedures during the remainder of their stay.

The severity of the burn was examined on admission by the attending physician, who took into account the appearance of the wound, capillary refill, and the sensory function of wounds, and was recorded in detail on a Lund & Browder chart. Patients were treated according to a fixed protocol (previously described), which included: standard ventilation when needed^18^; parenteral fluids^19^; early enteral nutrition, delirium prevention^20^; early excision and grafting^21^; and wound care every second day either by dressing change only or debridement.

### Statistics

Descriptive data have been presented as median and 25th–75th centiles (IQR) or n (%), OR and values of area under the curve (AUC) have been presented with (95% CI). Probabilities of less than 0.05 were accepted as significant. Distribution was tested with the Lilliefors test. The significance of the differences between groups was assessed using the Mann Whitney U, and the chi-squared tests. Logistic regression (multivariable) was used to assess the effect of age, burn size (TBSA%), EMR, _a_SOFA, _a_Lactate, and _a_NLR) on mortality (survivors = 0, non survivors = 1). Receiver operating characteristic curves (area under the curve) (ROC AUC) were calculated after multivariable logistic regression. The models were compared pairwise with the chi-squared test (roccomp). Specificity, positive and negative predictive value, and proportion correctly classified were calculated post hoc (estat classification) and the Hosmer–Lemeshow (estat gof, group(10)) test and Brier score (brier) were used for assessment of model calibration. Data were analysed with the help of STATA (STATA v12.0, Stata Corp. LP, TX, USA).

### Ethics approval and consent to participate

All methods were carried out in accordance with relevant guidelines and regulations. As the study design was retrospective no experimental protocols were used. No children under 18 were included. The results were not presented in the individual level. The Regional Ethics Review Board for these reasons did not mandate individual consents from the patients included in the study. The study was approved by the Regional Ethics Review Board in Linköping (No. 2013/341-31).

## Results

### Demographics

A total of 222 patients were included in the study, of whom 148 (67%) were male, median (25th–75th centiles) age was 55.0 (38.0 to 69.0) years, TBSA% burned was 24.5 (13.0 to 37.2), Baux score was 81.8 (62.0 to 99.0), and crude mortality 17% (Table 1). The model with age and burn size showed an AUC of 0.906 (95% CI 0.857 to 0.955), (n = 222), (Table 2).

**Table 1:** Description of the patients.

	All	Survivors	Non survivors	*p*
No. of patients	222	185	37	
Sex, male	148 (66.7)	127 (68.6)	21 (56.8)	0.16
Age, years	55.0 (38.0–69.0)	52.0 (37.0–66.0)	69.0 (63.0–74.0)	< 0.001
Total burn size, BSA%	24.5 (13.0–37.2)	21.5 (12.5–32.5)	43.0 (32.0–63.0)	< 0.001
Superficial dermal burns, BSA%	2.0 (0.0–8.0)	2.0 (0.0–8.5)	0.0 (0.0–3.1)	0.007
Deep dermal burns, BSA%	5.5 (1.0–16.1)	5.3 (1.0–15.3)	11.0 (1.5–20.5)	0.44
Full thickness burns, BSA%	4.8 (0.0–16.8)	3.5 (0.0–11.2)	27.0 (7.0–45.5)	< 0.001
Admission, days after injury	0.4 (0.3–0.7)	0.5 (0.3–0.8)	0.4 (0.2–0.5)	0.01
Duration of hospital stay, days	32.5 (17.0–50.0)	34.0 (20.0–51.0)	6.0 (1.0–23.0)	< 0.001
Intensive care period, days	12.5 (2.9–29.8)	13.2 (3.6–29.8)	5.7 (1.1–23.7)	0.06
Baux score	81.8 (62.0–99.0)	75.5 (59.6–91.0)	113.0 (106.0–130.0)	< 0.001
Estimated mortality risk*	0.08 (0.04–0.16)	0.07 (0.03–0.12)	0.24 (0.10–0.40)	< 0.001
Admission SOFA score	5.0 (3.0–8.0)	5.0 (2.0–7.0)	8.0 (6.0–10.0)	< 0.001
Lactate mmol/L	1.4 (0.9–2.2)	1.3 (0.9–1.8)	2.4 (1.9–4.2)	< 0.001
Neutrophil to lymphocyte ratio	9.72 (5.38–16.16)	8.75 (5.19–14.67)	12.65 (7.46–18.64)	0.04
Neutrophil count × 10*9/L	13.5 (9.0–17.5)	12.8 (8.9–16.4)	16.1 (9.7–21.5)	0.02
Lymphocyte count × 10*9/L	1.4 (0.9–1.9)	1.4 (0.9–1.9)	1.3 (0.8–1.9)	0.55

Data are presented as median (25th–75th centiles) or n (%). BSA, body surface area. SOFA score, Sequential organ failure assessment score.

*EMR SAPS 3.

**Table 2:** Nine models for mortality, eight are compared to the first model (age and burn size).

	Coefficient	*p*	OR (95% CI)	R^2^	n	AUC (95% CI)	Chi squared	*p*
Age, years	0.10	< 0.001	1.10 (1.06 to 1.15)	0.41	222	0.906 (0.857 to 0.955)		
Burn size, BSA%	0.08	< 0.001	1.08 (1.05 to 1.11)					
Constant	− 10.46	< 0.001						
Estimated mortality risk	5.04	< 0.001	155.04 (18.99 to 1265.75)	0.12	222	0.786 (0.711 to 0.860)	7.85	0.005
Constant	− 2.48	< 0.001						
_a_SOFA score	0.28	< 0.001	1.33 (1.18 to 1.49)	0.14	222	0.758 (0.677 to 0.839)	10.3	0.001
Constant	− 3.51	< 0.001						
_a_Neutrophil to lymphocyte ratio	0.03	0.103	1.03 (0.99 to 1.07)	0.03	160	0.624 (0.515 to 0.733)	16.97	< 0.001
Constant	− 1.90	< 0.001						
_a_Lactate	0.86	< 0.001	2.35 (1.65 to 3.37)	0.22	188	0.818 (0.740 to 0.895)	2.74	0.098
Constant	− 3.37	< 0.001						
Age, years	0.08	0.000	1.09 (1.04 to 1.13)	0.43	222	0.913 (0.867 to 0.959)	0.76	0.382
Burn size, BSA%	0.08	< 0.001	1.08 (1.05 to 1.11)					
Estimated mortality risk	2.44	0.07	11.50 (0.81 to 163.52)					
Constant	− 10.01	< 0.001						
Age, years	0.10	< 0.001	1.11 (1.06 to 1.16)	0.47	222	0.926 (0.882 to 0.970)	2.91	0.088
Burn size, BSA%	0.08	< 0.001	1.08 (1.05 to 1.11)					
_a_SOFA score	0.25	0.002	1.29 (1.10 to 1.51)					
Constant	− 12.43	< 0.001						
Age, years	0.09	< 0.001	1.09 (1.05 to 1.14)	0.37	160	0.888 (0.825 to 0.951)	0.04	0.848
Burn size, BSA%	0.07	< 0.001	1.07 (1.04 to 1.11)					
_a_Neutrophil to lymphocyte ratio	0.01	0.73	1.01 (0.97 to 1.04)					
Constant	− 9.64	< 0.001						
Age, years	0.11	< 0.001	1.11 (1.06 to 1.17)	0.51	188	0.938 (0.898 to 0.979)	5.53	0.019
Burn size, BSA%	0.07	< 0.001	1.07 (1.04 to 1.10)					
_a_Lactate	0.90	0.001	2.47 (1.48 to 4.10)					
Constant	− 12.50	0.000						

Logistic regression, burn centre non survivors coded 1, survivors 0. R^2^ is the Pseudo R^2^. AUC, area under the curve (under the receiver operating characteristic curve). Chi squared p is calculated pairwise on the difference between the first model (age and burn size) and each of the other models (no correction). BSA, body surface area. _a_ = admission. SOFA score, Sequential organ failure assessment score.

### Simplified Acute Physiology Score 3; Estimated Mortality Risk (EMR)

Median (25th–75th centiles) EMR was 0.08 (0.04 to 0.16) for all patients, 0.07 (0.03 to 0.12) for survivors, and 0.24 (0.10 to 0.40) for non-survivors (*p* < 0.001), (Table 1). AUC for the EMR (simple) model was 0.786 (95% CI 0.711 to 0.860), (n = 222), (Table 2).

### Admission Sequential Organ Failure Assessment (_a_SOFA)

Median (25th–75th centiles) _a_SOFA score was 5.0 (3.0 to 8.0) for all patients, 5.0 (2.0 to 7.0) for survivors, and 8.0 (6.0 to 10.0) for non-survivors (*p* < 0.001), (Table 1). For a detailed SOFA scores presentation, see Supplementary file 1: Table S1. AUC for the _a_SOFA (simple) model was 0.758 (95% CI 0.677 to 0.839), (n = 222), (Table 2).

### Blood lactate concentration at admission (_a_Lactate)

**Figure 2:**
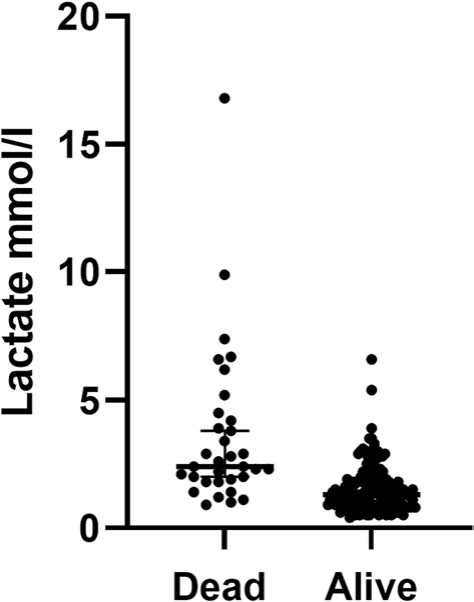
Lactate levels at admission among the patients that died and survived. Data as individual plots, lines = median and 25th–75th centiles.

Median (25th–75th centiles) _a_Lactate (mmol/L) was 1.4 (0.9 to 2.2) for all patients (n = 188), for survivors 1.3 (0.9 to 1.8), (n = 155), and non-survivors 2.4 (1.9 to 4.2), (n = 33), (*p* < 0.001), (Table 1 and Fig. 2). AUC for the _a_Lactate (simple) model was 0.818 (95% CI 0.740 to 0.895), (n = 188), (Table 2).

### Neutrophil to Lymphocyte Ratio at admission (_a_NLR)

Median (25th–75th centiles) _a_NLR was 9.72 (5.38 to 16.16) for all patients (n = 160), 8.75 (5.19 to 14.67) for survivors, (n = 131) and 12.65 (7.46–18.64) for non-survivors (n = 29), (*p* < 0.04), (Table 1). AUC for the _a_NLR (simple) model was 0.624 (95% CI 0.515 to 0.733), (n = 160), (Table 2).

### Model constructs (Logistic regression)

#### Baux score

The prediction model with age and burn size (TBSA%) was chosen for further analyses and comparisons as it resulted in the highest AUC value (Table 2). Other variants of the burn size variables (partial thickness; full thickness) were also tested together with age and the results were similar (data not shown).

#### EMR

Adding EMR to the model with age and burn size (TBSA%) resulted in a 0.007 higher AUC value but the difference was not significant (Table 2).

#### _a_SOFA

Adding _a_SOFA to the model with age and burn size (TBSA%) resulted in a 0.020 higher AUC value, but the difference was not significant (Table 2).

#### _a_Lactate

Adding _a_Lactate to the model with age and burn size (TBSA%) resulted in a significantly higher AUC value, increasing with 0.032 to 0.938 (95% CI 0.898 to 0.979), (n = 188), (*p* = 0.019), (Table 2).

#### _a_NLR

Adding _a_NLR to the model with age and burn size (TBSA%) resulted in a 0.018 lower AUC value, but the difference was not significant, (n = 160), (Table 2).

### Further combinations beyond age, burn size, and _a_Lactate

**Figure 3:**
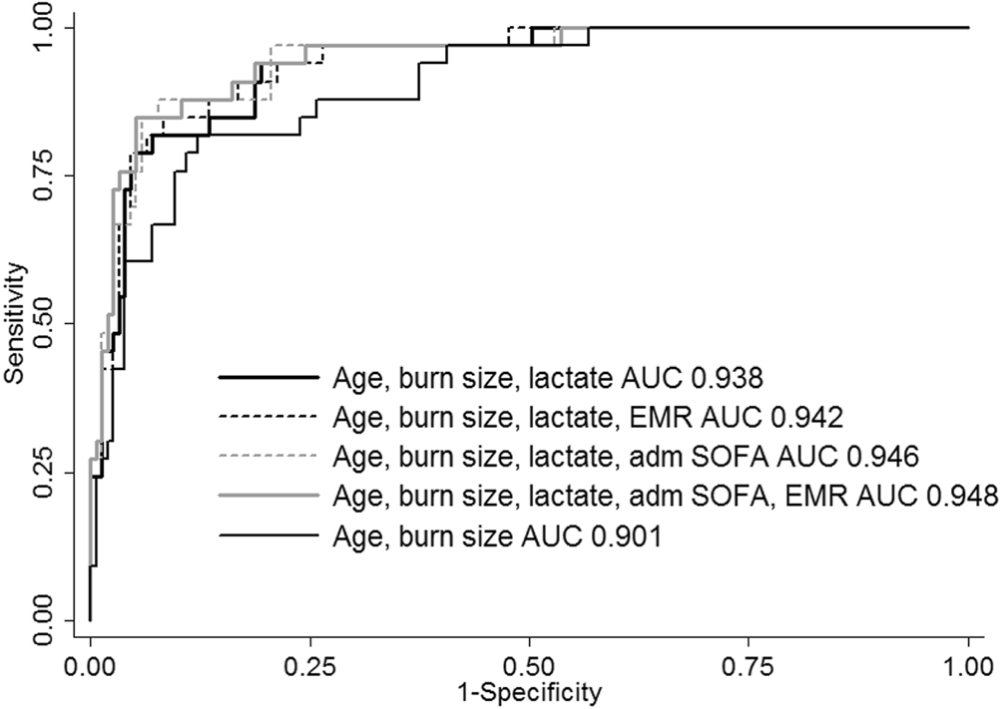
Receiver Operating Characteristic (ROC) area under the curve (AUC) for the combined models. All models were stronger than that with age and burn size (AUC 0.901) (*p* < 0.05) (Supplementary file 1: Table S2), and none of the models with the highest AUC values (0.942 to 0.948) were significantly stronger than that with age, burn size, and lactate (AUC 0.938) n = 188 (see Table 3 for more details).

Adding EMR, _a_SOFA, and _a_NLR to the combination of age, burn size, and lactate did not further improve the mortality prediction (not significant). Neither did the attempt to combine all remaining* factors (age, burn size, EMR, and _a_SOFA) (Table 3, Fig. 3**)**. (*At this stage, we had chosen to not include _a_NLR in additional combinations for two reasons, the contribution of this variable was not significant in any of the previous steps, and to avoid further attrition due to missing data).

**Table 3:** ROC-AUC for mortality and pairwise comparison to the model with age, burn size, and lactate.

	AUC (95% CI)	X^2^	p*	Brier score	H–L X^2^	p	Sensitivity	Specificity	Correctly classified
Age, burn size, _a_Lactate	0.938 (0.898 to 0.979)			0.070	7.31	0.50	57.6	96.1	89.4
Age, burn size, _a_Lactate, _a_SOFA	0.946 (0.906 to 0.985)	1.70	0.19	0.064	10.3	0.25	66.7	96.8	91.5
Age, burn size, _a_Lactate, EMR	0.942 (0.903 to 0.981)	0.78	0.38	0.069	7.3	0.50	60.6	96.8	90.4
Age, burn size, _a_Lactate, _a_SOFA, EMR	0.948 (0.909 to 0.988)	2.75	0.10	0.064	10.1	0.26	69.7	97.4	92.6

*Pairwise comparison to the first model, no correction, n = 188. SOFA, Sequential organ failure assessment score. EMR, Estimated mortality risk. H–L, Hosmer–Lemeshow test.

**Figure 4:**
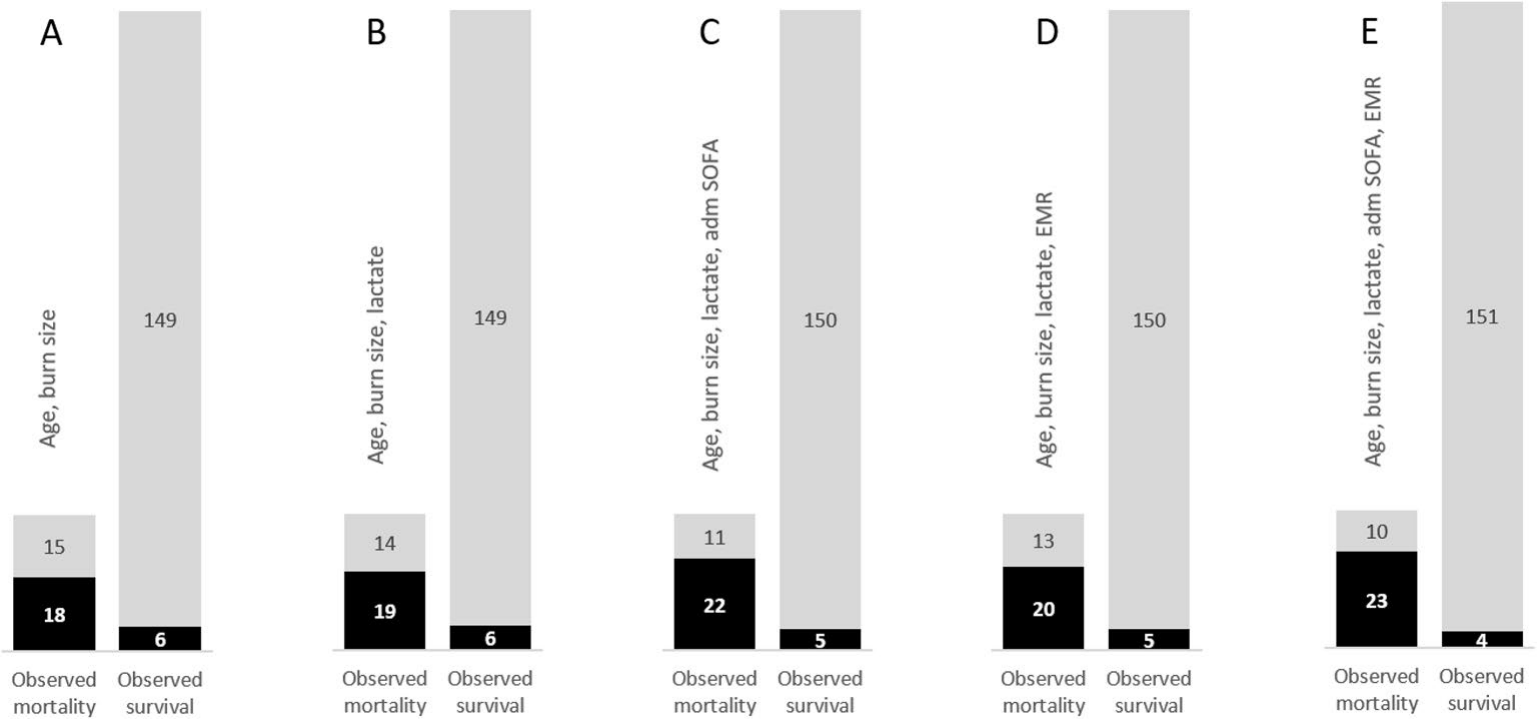
Observed and predicted mortality by five models. Observed mortality was 33 patients and observed survival was 155 (calculated on the 188 patients who had blood lactate taken at admission). The first two bars on the left side (**A**) show that the variables age and burn size, predicted 18 true positive and 149 true negative. (**B**) The model with age, burn size, and lactate, predicted 19 true positive. (**C**) That with age, burn size, lactate, and admission SOFA, predicted 22 true positive and 150 true negative. (**D**) The model with EMR instead of SOFA, predicted 20 true positive. (**E**) The combination of the previous variables resulted in 23 true positive and 151 true negative. Predicted mortality = black. Predicted survival = grey.

Figure 4 shows the number of patients by observed and expected, mortality and survival, according to the post regression classifications done for five models: that with age and burn size and the four models presented in Table 3.

Further, adding calendar year to the combined mortality prediction model with all remaining factors showed that the factor calendar year (continuous variable) was significant (*p* = 0.02) with a coefficient of − 0.28 (95% CI − 0.53 to − 0.04), (n = 188) (Supplementary file 1: Table S3).

## Discussion

This study was set to examine if the well-known combination of age and burn size (often calculated as the Baux score and used as the major mortality prediction model for burns) could be further improved when using mortality predictions developed for the general critical care setting such as SAPS3 instead, or by applying admission _a_SOFA, _a_Lactate, or the _a_NLR. It was also examined if these measures (SAPS 3, _a_SOFA, _a_Lactate or _a_NLR), used alone or in combinations, would enhance mortality prediction for burns critical care settings. The data show that neither SAPS 3, _a_SOFA, _a_Lactate or _a_NLR alone were as good as the combination of age and burn size. A new and important finding in this study is that addition of _a_Lactate did improve mortality prediction when added to these variables, increasing the AUC from 0.90 to 0.94. This improvement was not only significant in a relatively small study group, but was also clinically important as the sensitivity of the model was increased and as the AUC value was increased more than 0.03 points^9^. Thereafter no further improvement of the mortality prediction could be established by adding any more variables or combining them. It was interesting to find, when examining the effect of time by including treatment year in the final model, that an improvement in survival (25% per year) could be shown despite the relatively small study group, which underlined the usefulness of the model when applied to data gathered from a single centre, which is in the smaller range when examined in the international perspective^9,22^.

The SAPS 3 prediction model is well known and considered to be a gold standard for ICU mortality predictions^23^. It was interesting to find that the mortality prediction of the SAPS3 model was less than anticipated, i.e. 0.79 (AUC). This is also less than normally registered for general ICU patients in, for example the Swedish Intensive Care Registry^24^, or in other international settings^25,26^ where values are often in the range close to 0.85^24,26^. The reason for this lower value is unclear and may depend on the fact that comparatively few burns were included in the initial cohort used for the development of SAPS3^11,27^. It needs also to be emphasised that it may be an effect of chance as the confidence interval for the SAPS 3 in this study was rather wide (0.71 to 0.86) due to limited number of observation. Unfortunately, we were unable to improve the mortality prediction by adding the SAPS3 value to the Baux score variables. As SAPS 3 scores included the physiological derangement at admission together with a comorbidity estimate, it was hypothesised that the mortality prediction would improve. The reason it did not may be because the ages of the burns patients were rather low, with few comorbidities, and that, especially if there was an early admittance after the injury, the physiological derangements could have been low. This is supported by the low EMR registered (0.08 for all patients and 0.24 for those dying) compared with patients treated in general intensive care^24,28^. Thus, for burn care, the variables of the Baux score still remain the mainstem for mortality predictions. We only used one burn specific model as the basis to analyse the effect of the other variables in the present study as we have found that many of the burn specific models show similarly high predictive values, the ROC AUC values were well above 0.95 when calculated on the whole cohort of burns regardless of injury severity. Further, we chose to use the Baux score as its predictive value was among the strongest in our previous study^3^.Admission SOFA has been claimed to be a relevant predictor of mortality^29,30^. It is also well known that patients with large burns have more pronounced organ dysfunction at admittance^31^, albeit not all of those in a cohort that have a mean TBSA of 28% and a Baux score of 81.9, as in this study. The limited number of deaths may further contribute to the non-significant findings. Although the mortality prediction capability of _a_SOFA alone (simple regression, AUC 0.76) could be classified as fair it was also limited when added on to the variables of the Baux score.

Elevated lactate values have been shown to influence mortality repeatedly and it was a pre-study hypothesis of this paper also^12,32,33^. We think that elevated lactate values during the fluid resuscitation period can be attributed mainly to local or general tissue hypoperfusion^33^ even when traditionally used endpoints for fluid resuscitation have been reached^34–36^. Somewhat astonishingly, however, was the restricted number of patients with increased lactate at admission and also, that the range of these _a_Lactate values was limited (Fig. 2), i.e., most of them were close to or within reference values. Similar figures have, however, been presented previously. Kamoltz et al. reported that more than half (88/166) of their study group (mean TBSA burned was 30%) had _a_Lactate values of less than 2 mmol/l^32^. Cochran et al. reported somewhat higher values in a study group with larger burns (TBSA 42%), _a_Lactate (mean) was 3 mmol/l among survivors (n = 97) and 4.2 among those who died (n = 21)^12^. Despite this relatively small increase in _a_Lactate it still contributed to an increased mortality precision prediction beyond that of the variables of Baux score, and this increase resulted in a stronger (*p* = 0.04) model than by adding EMR (SAPS 3) instead of _a_Lactate. The AUC value with _a_Lactate combined with age and size of burn was higher also than that with the use of _a_SOFA instead, but not significantly so (*p* = 0.18). We think that the magnitude of the effect of _a_Lactate is clinically relevant.

Admission Neutrophil to Lymphocyte ratio has been found to be predictive for mortality estimates^14^. This may be anticipated to be so particularly as it has been shown that very high leucocyte counts can be registered early after burns and they correlate to the size of the burn, when there is a flood of leucocytes from the bone marrow into the central circulation^37^. Also, this estimate may then be claimed as an outcome measure of the burn trauma and can be hypothesised to predict mortality. However, in this study despite the significant difference (*p* < 0.04) between the _a_NLR between those dying and survivors, 12.6 and 8.8, respectively, it was not found to affect the mortality prediction on its own, nor did it significantly improve the prediction of mortality when added to the variables of the Baux score. The fact that the burn size was bigger (median 55% TBSA) and the (median) NLR on day 1 was approximately 15 in the cohort studied by Hu et al.^14^ could explain why we had different results from the regression models, although other studies with burn size and NLR values more similar to ours have also shown NLR to be an independent factor for mortality^38,39^. It is possible that our time frame (within 48 h) of “admission” NLR increased the variation so much that the actual effect was blurred. Hu et al. showed a marked decrease in NLR over the first three days after injury, from approximately 15 on day 1, to 10 and 9 on the second and third days, respectively^14^ which is similar to the figures presented by Qiu et al. who also studied more severe burns (53% TBSA%) with NLR levels of 15 on day 1. In their study, however, it was the NLR on day three that was significant (independent factor for 90 day mortality)^40^.

### Clinical perspective

Baux score remains the most important method of mortality prediction for burns. A new and important finding in this paper has been that lactate at admission can further increase the precision of the mortality prediction and in a burn cohort the prediction levels reach an AUC of 0.94. This is particularly useful for the benchmarking of burns care and the assessment of the quality of burns care work. There is also value from a clinical perspective because specificity is high, but there is still a shortcoming, which is the limited number of deaths in the database that has resulted in decreased sensitivity (predicting who will die amongst those who are dying)^3^. Another interesting clinical observation in this study was the improvement of care (decreasing mortality) over time that amounted to about 25% per year (relative risk, OR 0.75), which corresponded to a total reduced risk-adjusted mortality of about 94% over the ten years (e^(10*(− 0.28)) = 0.06), with a range (95% CI) between 33.0% and 99.5%. In a previous study, when we included all burns (not only those who needed critical care) admitted during the period 2000–2015, we found that mortality decreased over time without an increase in the daily use of resources^4^.The conclusion at that time was that improvements in quality had been achieved within our multidisciplinary routines for the care of patients.

The aim of the present study was, however, not to study changes in mortality over time. We used the variable calendar year in the regression to examine if the combined model was comprehensive enough to make the remaining variability of mortality by calendar year small enough to show a significant effect over time. The underlying assumption is that there is a continuous progress with advances in multiple medical treatments, which is anticipated to further improve survival after burns^6^. The positive finding (improved mortality) is thus merely an indication of a comprehensive model, it should not be interpreted as a statistical fact or evidence.

Furthermore, the data can be generalised to other settings outside Scandinavia, as the results are in line with previous studies that have come from different parts of the world using the variables of the Baux score as a mortality prediction model^3^. This applies to the _a_Lactate data as well, which are relevant to what has been shown for the resuscitation period in the treatment of major burns internationally^12,32^.

### Limitations/strength

This study was based on data from a single centre in a high-income country and with strict measures for the management of burns. Thus, this model may need local validation in other centres with different management strategies. The relatively small study group is a limitation, especially considering the small number of non-survivors which was further decreased due to missing data of biomarkers. The models that included _a_Lactate were calculated on 33 non-survivors while the models that included _a_NLR were calculated on 29 non-survivors. The low p value (0.10) for _a_NLR in the simple regression suggests the possibility that it could have shown a significant effect if there had been no missing data (Table 2). The combination with age, burn size, and _a_NLR, however, indicated that the _a_NLR was not an independent factor in the present study (Table 2). In order to further investigate if the negative results regarding _a_NLR could be explained as an effect of attrition we repeated the regressions using only the 160 patients who had _a_NLR values (no data shown). All variables except for _a_NLR and EMR showed a significant effect in those regressions, which was similar to the results presented in the current tables (and supplementary tables).

### Future perspectives

It would be advantageous if the mortality prediction model could be further refined. One area that has not been addressed specifically is early kidney affection, which has a significant mortality prediction value, especially when seen later during care^41^. This may be a future area of interest. The kidney factor in the SOFA score may be of value but this variable is rather crude and insensitive in the present scoring setting. Simple logistic regression has shown a coefficient of 0.45 (*p* = 0.001) but it was not an independent factor after adjustment for age and burn size (*p* = 0.10), and the p-value for the kidney factor was increased (0.46) when _a_Lactate also was added (further data not shown).

## Conclusion

This study shows that amongst the attempts made to improve the Baux score mortality prediction (i.e. SAPS3) admission SOFA score, lactate concentration, and the Neutrophil to Lymphocyte ratio, only admission lactate was found to be clinically useful, improving the AUC of the Baux score from 0.90 to 0.94. More studies are needed to further improve mortality prediction, especially the sensitivity of the model.

## Supplementary Information

Supplementary Tables.

## Data Availability

The dataset used and/or analysed during the current study are available from the corresponding author on reasonable request.
