# Supplementary material for: Addition of admission lactate levels to Baux score improves mortality prediction in severe burns
Source: Sci Rep. 2021 Sep 10;11:18038. doi: 10.1038/s41598-021-97524-9 (PMC8433150; doi:10.1038/s41598-021-97524-9)
Supplement: Supplementary file 1 — Supplementary Tables. [file 41598_2021_97524_MOESM1_ESM.pdf]

Addition of admission lactate levels to Baux score improves mortality prediction in severe burns

Ingrid Steinvall PhD[1,2*], Moustafa Elmasry MD, PhD[1,2], Islam Abdelrahman MD, PhD [1,2], Ahmed El-Serafi MD, PhD [1,2,3], Folke Sjöberg MD, PhD[1,2,4]

[1]Department of Hand Surgery, Plastic Surgery and Burns, Linköping University, Linköping, Sweden.

[2]Department of Biomedical and Clinical Sciences, Linköping University, Linköping, Sweden.

[3]Medical Biochemistry department, Faculty of Medicine, Suez Canal University, 41522, Ismailia, Egypt.

[4]Department of Anaesthesiology and Intensive Care, Linköping University, Linköping, Sweden.

Table S1 SOFA score by organ dimension recorded at admission and as maximum dysfunction score by organ

	All	Survivors	Non survivors	
Admission SOFA				
Respiratory	2 (0–3)	1 (0–2)	3 (2–3)	<0.001
Coagulation	0 (0–0)	0 (0–0)	0 (0–1)	0.29
Liver	0 (0–1)	0 (0–0)	0 (0–1)	0.08
Cardiovascular	3 (1–3)	1 (0.5–3)	3 (1–4)	0.001
Renal	0 (0–1)	0 (0–0)	1 (0–3)	<0.001
CNS	0 (0–1)	0 (0–1)	0 (0–3)	0.11
Maximum SOFA				
Respiratory	3 (2–4)	3 (2–4)	3 (3–4)	0.37
Coagulation	1 (0–2)	1 (0–2)	2 (1–3)	0.002
Liver	0 (0–1)	0 (0–1)	2 (0–2)	<0.001
Cardiovascular	4 (3–4)	3 (1–4)	4 (4–4)	<0.001
Renal	1 (0–3)	0 (0–2)	3 (3–4)	<0.001
CNS	2 (0–3)	2 (0–3)	4 (1–4)	<0.001

Data are presented as median (25th–75th centiles). SOFA score, Sequential organ failure assessment score.

Table S2 ROC-AUC for mortality and pairwise comparison to the model with age and burn size

	AUC (95% CI)	X^2	p*	Brier score	H-L X^2	p	Sensitivity	Specificity	Correctly classified
Age, burn size	0.901 (0.846 to 0.955)			0.085	1.5	0.99	54.6	96.1	88.8
Age, burn size, $_{a}$Lactate, $_{a}$SOFA	0.946 (0.906 to 0.985)	6.49	0.01	0.064	10.3	0.25	66.7	96.8	91.5
Age, burn size, $_{a}$Lactate, EMR	0.942 (0.903 to 0.981)	5.72	0.02	0.069	7.3	0.50	60.6	96.8	90.4
Age, burn size, $_{a}$Lactate, $_{a}$SOFA, EMR	0.948 (0.909 to 0.988)	7.22	0.007	0.064	10.1	0.26	69.7	97.4	92.6

*pairwise comparison to the first model, no correction, n=188. SOFA, Sequential organ failure assessment score. EMR, Estimated mortality risk. H-L, Hosmer–Lemeshow test.

Table S3 Logistic regression for mortality, the combined/final model

Combined, final model

	Coefficient	p	OR
Age, years	0.10	<0.001	1.10 (1.05–1.16)
Burn size, BSA%	0.06	<0.001	1.07 (1.03–1.10)
Lactate	0.83	0.002	2.29 (1.34–3.89)
Admission SOFA score	0.18	0.04	1.20 (1.01–1.44)
Estimated mortality risk	1.51	0.38	4.51 (0.16–130.69)
Constant	-13.30	<0.001	

Model pseudo R^2 0.54, p<0.001. AUC 0.948 (95% CI 0.909–0.988), n = 188

Combined, final model, with the factor calendar year

	Coefficient	p	OR
Age, years	0.11	<0.001	1.11 (1.05–1.18)
Burn size, BSA%	0.07	<0.001	1.07 (1.04–1.11)
Lactate	0.79	0.003	2.21 (1.32–3.71)
Admission SOFA score	0.26	0.009	1.30 (1.07–1.59)
Estimated mortality risk	2.57	0.167	13.07 (0.34–500.35)
Calendar year	-0.28	0.023	0.75 (0.59–0.96)
Constant	556.20	0.026	

Model pseudo R^2 0.58, p<0.001. AUC 0.960 (95% CI 0.930 to 0.990), n=188.

Chi squared 2.39, p=0.12 for the difference between the two models above, with and without the factor calendar year.
